# A Web-Based Auditory and Visual Emotion Perception Task Experiment With Children and a Comparison of Lab Data and Web Data

**DOI:** 10.3389/fpsyg.2021.702106

**Published:** 2021-08-18

**Authors:** Hisako W. Yamamoto, Misako Kawahara, Akihiro Tanaka

**Affiliations:** ^1^Tokyo Woman’s Christian University, Tokyo, Japan; ^2^Japan Society for the Promotion of Science, Tokyo, Japan

**Keywords:** online experiments, emotion perception, cognitive development, auditory perception, visual perception, vocal expression, facial expression

## Abstract

Due to the COVID-19 pandemic, the significance of online research has been rising in the field of psychology. However, online experiments with child participants are rare compared to those with adults. In this study, we investigated the validity of web-based experiments with child participants 4–12 years old and adult participants. They performed simple emotional perception tasks in an experiment designed and conducted on the Gorilla Experiment Builder platform. After short communication with each participant via Zoom videoconferencing software, participants performed the auditory task (judging emotion from vocal expression) and the visual task (judging emotion from facial expression). The data collected were compared with data collected in our previous similar laboratory experiment, and similar tendencies were found. For the auditory task in particular, we replicated differences in accuracy perceiving vocal expressions between age groups and also found the same native language advantage. Furthermore, we discuss the possibility of using online cognitive studies for future developmental studies.

## Introduction

The COVID-19 pandemic that began in 2020 has forced people to move much of their daily, face-to-face communication online. Psychological experiments are no exception. Many behavioral scientists had to stop their research and decide whether to postpone it or to move it online. Although many researchers have been trying to conduct studies remotely, sufficient examination of the validity of online developmental research is absent to date. In the present study, we introduce an online trial of perception tasks for children. We conducted a simple experiment featuring an auditory and a visual emotion perception task using video chat and an online experiment platform with children (4–12 years old) and adult participants. We then examined the validity of these data (online data) with the data from our previous, similar laboratory trial (Kawahara et al., 2021).

Even before the pandemic, online experiment research targeting adults was becoming popular due to its advantages. Unlike laboratory experiments, in which participants tend to be limited to residents around universities (e.g., Henrich et al., 2010), in online studies researchers can recruit participants without geographical constraints. Moreover, online experiments pair well with crowdsourcing services. Such services enable researchers to collect large amounts of data at low costs within a short time (Stewart et al., 2017). Such advantages have led many cognitive psychology researchers to adopt data collected through online experiments (e.g., Mills and D’Mello, 2014; Shin and Ma, 2016; Laeng et al., 2018; Lavan et al., 2018; McPherson and McDermott, 2018; Carbon, 2020).

However, can online experiments ensure the validity and quality of the data they generate? To answer this question, some studies have compared online cognitive experiment data with laboratory experiment data and reported their success in replicating results (e.g., Crump et al., 2013; Simcox and Fiez, 2014; de Leeuw and Motz, 2016). Previous studies have also demonstrated some disadvantages of online studies. One such problem is high dropout rates (Reips, 2002; Zhou and Fishbach, 2016). Moreover, even when participants remained until the end of the experiment, some of them, known as “satisfiers,” might not devote the cognitive effort in the tasks (Miura and Kobayashi, 2016). In addition to considering issues with online participants, we should consider the variety of their environments. In most of online studies, participants use their own devices. For this reason, while the validity of online data has been ensured for within-subject designs (e.g., Semmelmann and Weigelt, 2017), the case for between-subject design has not been clearly made. These factors may lead to greater variance in web experiments compared to lab experiments (Germine et al., 2012). Thus, while Internet-based experiments are easy to participate in, there may be some problems due to this ease (see Paolacci and Chandler, 2014).

Now then, what is the situation with online research for child participants? We were able to find some trials and projects that shifted developmental research online. For example, Tran et al. (2017) tried to move an infant study online by recruiting participants through Amazon Mechanical Turk. They measured the length of time that 5–8-month-olds remained looking at various stimuli and reported success in capturing changes in their attention depending on the stimulus presented, even in an online data collection environment. Concerning behavioral measures, Klindt et al. (2017) reported that the large amount of data they collected from online participants revealed changes in cognitive skills (e.g., working memory, false belief, etc.) over the human lifespan. They collected the data through the BRAiN’US online platform for smartphones, and participants also included children (participants ranged from 5 to 85 years old). However, their study did not focus on the validity of online experiments with children, nor did it compare their data with lab data; rather their aim was to obtain a large dataset from a wide range of participants. More recently, Nussenbaum et al. (2020) investigated the decision-making strategies of participants aged 8–25 during an online task. They compared their results with data from previous lab experiments and were able to replicate age-related changes in strategy even in the online experiment. Moreover, some new online platforms for child research, such as Lookit (Scott et al., 2017), Discoveries Online (Rhodes et al., 2020), and Childlab (Sheskin and Keil, 2018) have been developed with the aim of enabling participation in remote studies for children who are not able to easily travel to a laboratory.

These studies notwithstanding, less remote developmental research is being conducted than remote cognitive studies targeting adults. Why have developmental researchers hesitated to choose online research to pursue their research questions? The lack of online developmental research may be caused by the following difficulties. First, it is difficult for participants to form a rapport with the experimenter during online experiments. A rapport is important for ensuring that child participants are as relaxed as possible while engaging in tasks. Second, we cannot always check whether a participant is really a child (and not an adult), and participant age is a critical factor in developmental research. Third, differences in performance between different aged participants may be difficult to observe because experiments with a between-subject design are not considered suitable for online research. However, as the pandemic continues, the benefits of online developmental research may surpass such disadvantages if we can ensure the data is valid. Thus, it is imperative to accumulate data from online developmental studies featuring various online tasks to determine its suitability for use in future research.

In this study, we report on our attempt at moving an experiment involving children’s perception tasks online. Our experiment consisted of video chat communication, and main tasks were controlled through the online experiment platforms. First, the experimenter communicated with the child participants and their parents via Zoom^1^ to check the child’s participation and to build rapport with them. Next, the experimenter guided participants to the browser experiment webpage built with Gorilla Experiment Builder^2^,^3^ (Anwyl-Irvine et al., 2020) and instructed them to engage in two simple emotion perception tasks. To investigate the validity of the obtained data, we compared web data for each task lab data for very similar tasks (Kawahara et al., 2021). We hypothesized that this online experiment method would reduce the issues usually associated with an online experiment. Specifically, we predicted that participants would perform as well in the online experiment as in the lab experiment and that the accuracy of each task would not differ between web and lab data.

In the emotion perception tasks, participants were asked to judge emotions by watching dynamic facial expressions or listening to vocal expressions. We chose these tasks for our online developmental research for two reasons. First, the development of emotional perception has not been investigated in online research. Second, the emotional judgment task enables us to examine the effect of stimulus presented through a web browser on auditory (vocal expression) perception and visual (facial expression) perception independently. To compare web data with lab data for each modality, participants engaged in an auditory task judging emotions by listening to sounds only, and a visual task judging emotions by watching facial dynamics only (with no sound).

## Materials and Methods

### Participants

#### Web Data

The 36 children aged 4–12 years old (30 girls and 6 boys) and the 16 undergraduate or graduate students (age range: 18–29, *M age* = 21.63, 13 women and 3 men) participated in the experiment. Since one 5-years-old girl’s parent reported that she used built-in laptop speakers because her earphones did not fit her, her data for both tasks were excluded from the analysis. In the analysis, 4–8-year-old children were classified as the younger child group (*N* = 21, *M age* = 6.48 years old) and the 9–12-year-old children were classified as the older child group (*N* = 14, *M age* = 10.29 years old). Data were collected from undergraduate or graduate students to compare the data collected from children with data collected from adults.

Child participant data were collected during the online science event of the National Museum of Emerging Science and Innovation (Miraikan) in Tokyo, Japan. We recruited participants through the Miraikan web page and SNS services (Twitter, Facebook). This event was held from August to December 2020. Adult participants were recruited through a snowball-sampling method and the Crowdworks crowdsourcing service website.^4^

All participants spoke Japanese as their native language. All adult participants and parents of child participants were informed of the purpose of the study and gave informed consent in accordance with the Declaration of Helsinki by checking a box on the consent page during the browser experiment session.

#### Lab Data

We compared lab data from the unimodal session of our previous experiment (Kawahara et al., 2021) with our web data. Data collected from 179 children aged 5–12 years old (75 girls and 104 boys) and from 33 undergraduate or graduate students (age range: 18–32, *M age* = 22.39, 17 women, 16 men) were included in the analysis. Child participants’ lab data were collected during the science event held at the Miraikan in 2015. We recruited participants through the Miraikan web page. For data analysis, the 5–8 year-old children comprised the younger child group (*N* = 100, *M age* = 6.36), and the 9–12 year-old children comprised the older child group (*N* = 79, *M age* = 10.66). Adult participants were recruited using a snowball-sampling method. As with participants in the web experiment, all participants in the lab experiment spoke Japanese as their native language. Adult participants and parents of child participants gave written informed consent in accordance with the Declaration of Helsinki.

### Stimuli

#### Web Data

The auditory and visual stimuli used were based on the audiovisual stimuli originally used by Tanaka et al. (2010). These audiovisual stimuli (stimuli used as the “congruent condition” in their study) were short video clips featuring a speaker expressing anger or happiness through face and voice expression. The speakers were four women (two native Japanese speakers and two native Dutch speakers). In each video clip, each speaker speaks one of four utterances containing only emotionally neutral linguistic information, including Hello (Japanese, “*Hai, moshimoshi*”; Dutch, “*Hallo, dat ben ja*”) and Good-by (Japanese, “*Sayonara*”; Dutch “*Een goede dag*”); What is this? (Japanese, “*Korenani*”; Dutch “*Hey, wat is dit*?”); and Is that so? (Japanese, “*Sounandesuka*”; Dutch, “*Oh, is dat zo?*”). A total of 32 video clips [in two languages (Japanese and Dutch) × two emotions (angry and happy) × two speakers × four utterances] were used.

Auditory stimuli were created by turning off the images and adding a gray rectangle image of the same size. Visual stimuli were created by muting sounds. Auditory stimuli comprised 32 video clips with vocal expression information only. Visual stimuli comprised 32 video clips with facial expression information only. The resolution of each video clip was 640 × 480 pixels. In the web experiment, auditory and visual stimuli were encoded in MP4 files for web page presentation.

#### Lab Data

The web experiment stimuli and the lab experiment stimuli were almost same but differed in file format. In the lab experiment, the auditory stimuli files were in WAV format and the visual stimuli files in AVI format. Moreover, in the lab experiment auditory stimuli were presented with a blank, white display, and in web experiment a gray rectangle was displayed while the auditory stimuli were presented. The latter was to prevent web participants from becoming anxious due to watching a mere blank display in an experiment in which the experimenter is not present, unlike in a lab experiment.

#### The Validation of Stimuli

The validation of our stimulus set was verified in Kawahara et al.’s (2021) study, which investigated cross-cultural audiovisual emotion perception. Overall, there was no remarkable difference between Japanese and Dutch stimuli. For auditory stimuli, the average fundamental frequency (f0) was higher in Japanese than in Dutch for the happy voice stimuli (*z* = –3.36, *p* < 0.001), but not for the angry voice stimuli (Table 1). Considering that both Japanese and Dutch adult participants in Kawahara et al. (2021) responded to their ingroup voice stimuli more correctly than to their outgroup stimuli, this difference reflected their natural expressions in each culture. For visual stimuli, a certified FACS (Facial Action Coding System; Ekman and Friesen, 1978) coder coded all activated AUs during each stimulus. There was no difference in activated AUs except for AU17^5^ in angry faces (*z* = –3.00, *p* = 0.01) between Japanese and Dutch visual stimuli. Thus, the stimulus set was validated.

**TABLE 1:** The average fundamental frequency (f0) of auditory stimuli (Hz).

	Angry	Happy
Japanese	242.8	336.9
Dutch	233.1	261.4

### Apparatus

#### Web Data

Participants used their own earphones or headphones to listen to auditory stimuli and their own computers to watch visual stimuli and control the browser experiment program. We asked participants to use a computer monitor and earphones (or headphones) and recommended that they use the latest version of Google Chrome. We did not specify the models of the devices.

The resolution of participant displays ranged from 915 × 515 to 1920 × 1080. The participants’ used Windows (45 participants), Mac OS (3), Android (2), and iOS (1) operating systems and Google Chrome (27), Microsoft Edge (19), Microsoft Internet Explorer (4), and Safari (1) web browsers.

#### Lab Data

Researchers provided headphones (SONY MDR-ZX660) (used at a comfortable listening level) to present auditory stimuli and computers (Latitude 3540, Dell) to present visual stimuli and control the experiment program using Hot Soup Processor (Onion Software).

### Procedure

#### Web Data

**FIGURE 1:**
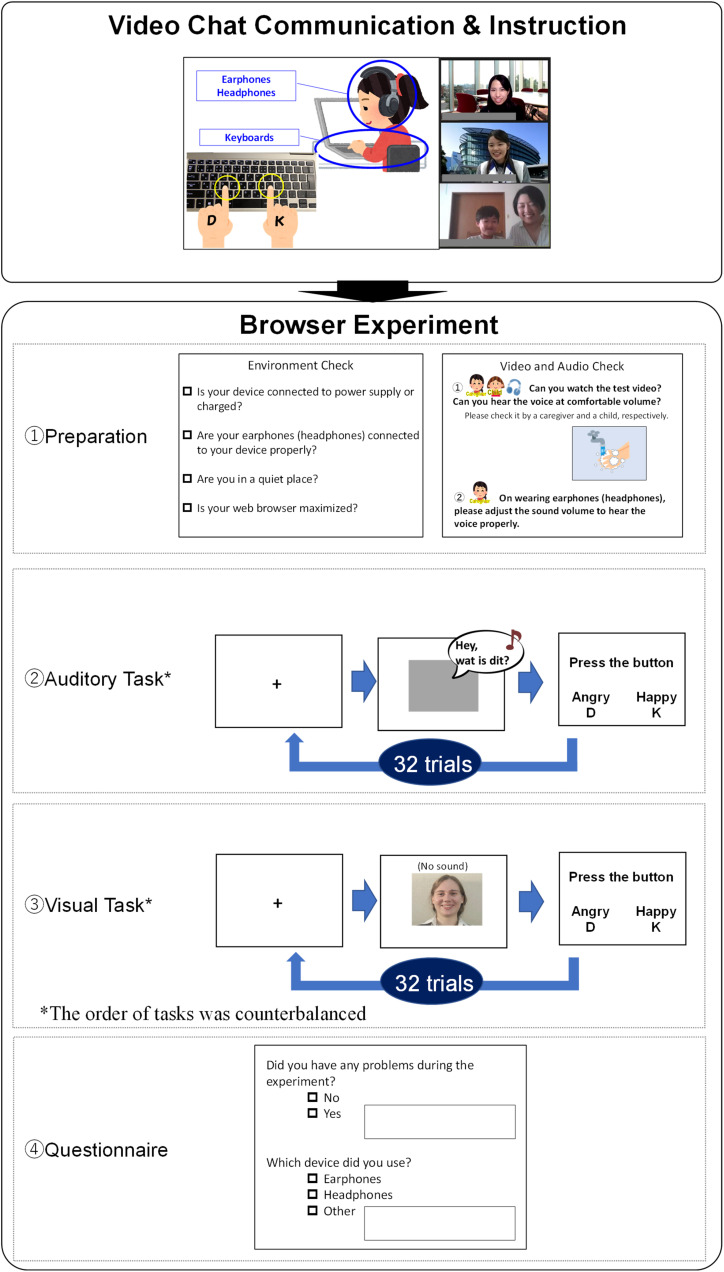
Experiment flow (The instructions and alternatives were written in Japanese in the actual experiment).

The flow of the procedure is shown in Figure 1. Before the experiment, child participants’ parents and adult participants received an instructions and documents file that included how to participate in the event and research brief. At the starting time, each participant and their parent joined the Zoom meeting room. The experimenter and the staff communicated with each participant using their web cameras and microphones to help participants relax. After a short communication, the experimenter provided attendees with instructions (e.g., not to click the web browser back button during the experiment, not to influence their children’s responses), checked that participants understood the positions of keys for response (D and K) on their keyboards, and guided them to the experiment web page by providing the URL link in the meeting room chatbox. After checking that each participant succeeded in accessing the experiment page, the experimenter instructed each participant to quit the meeting room to avoid low internet connection speeds during the experiment. They were also instructed to return to the same meeting room if they had any problems or reached the browser experiment’s final display.

In the browser experiment session, participants’ parents proceeded with the experiment by themselves following instructions on the display. We used Gorilla Experiment Builder to control the experimental program and collect data. The browser experiment session consisted of a preparation section, the auditory task, the visual task, and a questionnaire. In the preparation section, participants’ parents gave informed consent and indicated that environment requirements were met (sufficient device battery, headphones or earphones connection, environmental silence, web browser maximization) using checkboxes. Next, parents checked the sound volume with child participants, MP4 file playback, and keyboard operation following displayed instructions. After preparation, participants engaged in the auditory task and the visual task in each task section. The order of tasks was counterbalanced. At the beginning of each task section, participants watched a task instruction movie that included a simple speaking animation describing the task. The flow of each task is shown in Figure 1 (see panels 2 and 3, Auditory Task and Visual Task).

In the auditory task, participants were instructed to listen to a voice and judge whether the speaker was angry or happy. A fixation point was displayed at the center of the monitor for 500 ms, after which an auditory stimulus was presented. When the response alternatives written in hiragana characters^6^ were displayed, participants responded by pressing D or K keys (i.e., the allocation of response alternatives was counterbalanced). Five hundred ms after participant’s response, the next test trial began, for a total of 32 trials. In the visual task, participants were instructed to observe the face of a (muted) speaker and judge whether they were angry or happy. A fixation point was displayed at the center of the monitor for 500 ms, and each visual stimulus was presented successively. As with the auditory task, responses were indicated by pressing keys. 500 ms blank displays were inserted between trials, for 32 trials. For each task, the main trials were conducted following two practice trials.

After these tasks, in response to the questionnaire, participants’ parents reported problems during the experiment, whether participants had worn earphones, headphones, or had used other devices, whether parents had instructed their children to press a specific key during the main trials (“Did you ask your child to press any specific key during a task, for example, by saying ‘Press this key’?”); if they had any concerns, they were asked to fill out a form. According to the questionnaire, we confirmed that all participants included in the analysis had worn earphones or headphones, and that no parent instructed their child to press any specific key. None of parents reported any problems and concerns related with the tasks.

Adult participants similarly joined a Zoom meeting room before the browser experiment and received instructions. They were to proceed with the browser experiment by themselves and return to the same meeting room if they had any problems or had reached the last display in the browser experiment.

The procedure was similar to that of the child participants except that for adult participants the instructions were rewritten (e.g., converting some hiragana characters to kanji characters for readability^7^), and they were not asked a question about parents’ instruction (“Did you ask your child to press any specific key during a task, for example, by saying ‘Press this key’?”) in the questionnaire after the task.

#### Lab Data

**FIGURE 2:**
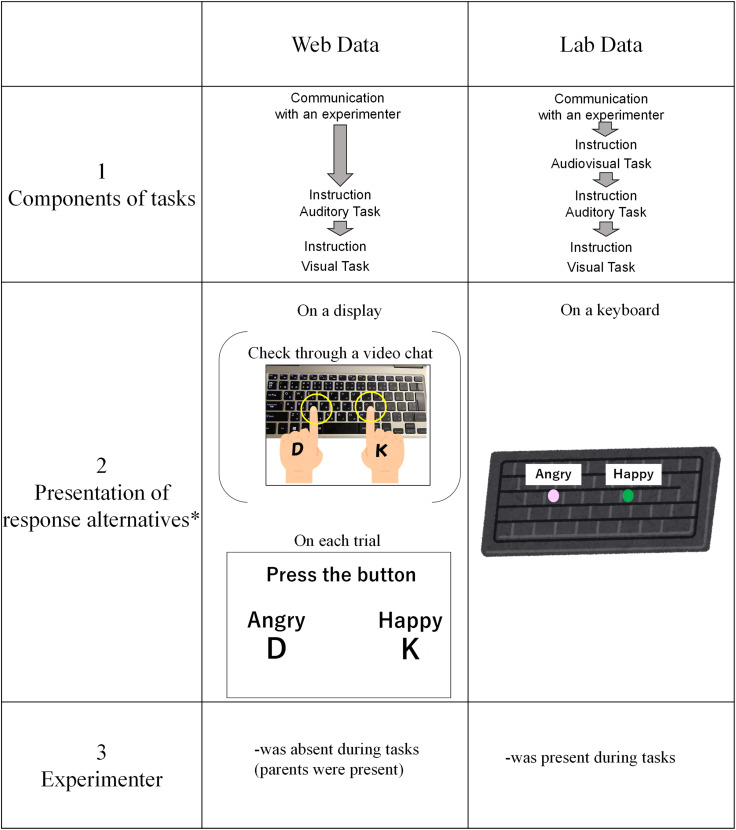
The differences in procedures between web and lab data.

The experiment was conducted in an experimental room at the Miraikan for the child participants and in an experimental room at the Tokyo Woman’s Christian University for the adult participants. The procedure was almost the same as with the web experiment but with three slight differences. First, in the (Kawahara et al., 2021) lab experiment, auditory and visual tasks were conducted after audiovisual emotional perception tasks in which participants judged speakers’ emotions after being presented with face and voice simultaneously. Participants in the web experiment did not engage in audiovisual emotion perception tasks like those in the lab experiment. We cannot rule out the priming effect in the lab data induced by the audiovisual stimuli that had been presented before. However, considering that the number of presentations of “angry” and “happy” stimuli was the same in the audiovisual emotion perception task, a response bias is not possible. Second, cards showing the alternatives (“angry” and “happy” written in hiragana characters) were put on a keyboard in the lab experiment; these alternatives were shown on the display in the web experiment. Third, the experimenter was physically present next to each participant and controlled the experiment program throughout the experiment session in the lab experiment. The presentation of stimuli was controlled using the Hot Soup Processor (Onion Software). These differences were summarized in Figure 2.

## Results

We calculated the rate of correct responses for each participant. Then, this rate was arcsine transformed to increase the normality of its distribution (accuracy). To investigate whether the experiment method affected different aged participants’ performance differently, we conducted a 2 (method: web, lab) × 3 (age group: younger child, older child, adult) × 2 (stimulus culture: Japanese, Dutch) mixed-factorial ANOVA on accuracy for each task.

### Auditory Task

**FIGURE 3:**
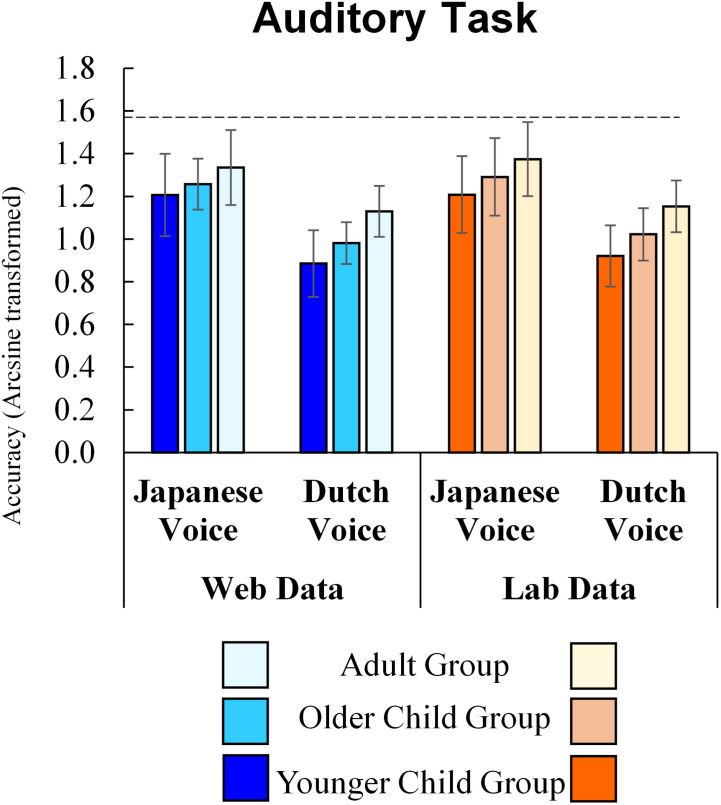
Auditory task Accuracy (Error bars indicate standard deviations and a dashed line indicates a perfect score).

The results for the auditory task are shown in Figure 3. In the auditory task, the main effects of method (*F*(1, 257) = 2.07, *p* = 0.151, η_p_^2^ = 0.008), the interaction of method and age group (*F*(2, 257) = 0.09, *p* = 0.917, η_p_^2^ = 0.001), of method and stimulus culture (*F*(1, 257) = 0.07, *p* = 0.789, η_p_^2^ < 0.001), and of the second-order interaction of method, age group, and stimulus culture (*F*(2, 257) = 0.22, *p* = 0.800, η_p_^2^ = 0.002) were not significant. Thus, the results of the auditory task using online tools were not significantly different from those of the lab experiment.

As for other factors, results showed significant main effects for age group (*F*(2, 257) = 31.56, *p* < 0.001, η_p_^2^ = 0.20). The *post hoc* analysis (Shaffer’s Modified Sequentially Rejective Bonferroni Procedure) revealed that the older child group of participants responded correctly to more stimulus than the younger child group and that adult participants responded correctly more often than younger and older child participants (*ps* < 0.001). The main effect of stimulus culture was also significant (*F*(1, 257) = 281.61, *p* < 0.001, η_p_^2^ = 0.52), showing that participants responded correctly to the Japanese voice more often than to the Dutch voice. Interaction between age group and stimulus culture was marginally significant (*F*(2, 257) = 2.89, *p* = 0.057, η_p_^2^ = 0.02). To check whether the impact of stimulus culture was different among age group, we conducted a simple main effect analysis. The simple main effect analysis also showed that all groups selected more correct answers in responses to the Japanese voice than in response to the Dutch voice (Younger child group: *F*(1, 119) = 157.56, *p* < 0.001; Older child group: *F*(1, 91) = 97.22, *p* < 0.001; Adult group: *F*(1, 47) = 59.11, *p* < 0.001). Moreover, a simple main effect of age was significant for both the Japanese voice (*F*(2, 257) = 9.01, *p* < 0.001) and the Dutch voice (*F*(2, 257) = 42.04, *p* < 0.001). *Post hoc* analysis (Shaffer’s Modified Sequentially Rejective Bonferroni Procedure) revealed that older child participants responded correctly to the Japanese voice more than the younger child group (*p* = 0.049), and that adult participants responded correctly more often than younger (*p* < 0.001) and older child participants (*p* = 0.034). A similar accuracy difference between age groups was observed with the Dutch voice. Older child participants responded correctly to the Dutch voice more often than the younger child group, and adult participants responded correctly more often than younger and older child participants (*ps* < 0.001). All age groups responded correctly more often to the Japanese voice than the Dutch voice, and accuracy with both voices increased with age.

To further examine the marginal interaction between age group and stimulus culture, we conducted a two-way ANOVA (method × age group) on the ingroup advantage. This was calculated by subtracting the accuracy of Dutch voices from that of Japanese voices. The main effect of age groups was marginally significant (*F*(2, 257) = 2.89, *p* = 0.057, η_p_^2^ = 0.02). *Post hoc* analysis (Shaffer’s Modified Sequentially Rejective Bonferroni Procedure) showed that the difference between younger children and adults was marginally significant (*p* = 0.051), suggesting that the ingroup advantage in younger children was more salient than that in adults. The differences between other pairs were not significant (younger child group – older child group: *p* = 0.149, older child group – adult group: *p* = 0.393). The main effects of method (*F*(1, 257) = 0.07, *p* = 0.789, η_p_^2^ < 0.001) and the interaction of method and age group (*F*(2, 257) = 0.22, *p* = 0.800, η_p_^2^ = 0.002) were not significant.

To further examine the effect of method on accuracy, we conducted a Bayesian repeated measures ANOVA on accuracy using JASP (2018) with default prior scales. Table 2 shows the inclusion probabilities and the inclusion Bayes factor (Clyde et al., 2011; van den Bergh et al., 2019). The inclusion Bayes factors reflect the average across possible models and reveal whether models with a particular predictor are more likely to have produced the observed data than those without. This approach is especially useful when the number of potential variables under consideration is large. The Bayesian ANOVA revealed that the BFinclusion values of the effect of method (BFinclusion = 0.165), the interaction effect between method and stimulus culture (BFinclusion = 0.111), the interaction effect between method and age group (BFinclusion = 0.069), and the second-order interaction of method, age group, stimulus culture, and age (BFinclusion = 0.004) were all small, supporting no effect of the difference between the web and lab experiments. Additionally, the data provide strong evidence for the effects of age group and stimulus culture (BFinclusions > 100), although they are not sufficiently informative to allow a strong conclusion about the effect of the interaction between age group and stimulus culture (BFinclusion = 1.095).

**TABLE 2:** Evidence for the presence of particular effects in the accuracy of the auditory task (Data averaged over all the models including/excluding a particular predictor).

	P(incl)	P(excl)	P(incl| data)	P(excl| data)	BFincl
Stimulus Culture	0.737	0.263	1.000	0.000	∞
Age Group	0.737	0.263	1.000	0.000	∞
Method	0.737	0.263	0.316	0.684	0.165
Stimuli × Age Group	0.316	0.684	0.336	0.664	1.095
Stimuli × Method	0.316	0.684	0.049	0.951	0.111
Method × Age Group	0.316	0.684	0.031	0.969	0.069
Stimuli × Method × Age Group	0.053	0.947	> 0.001	1.000	0.004

### Visual Task

**FIGURE 4:**
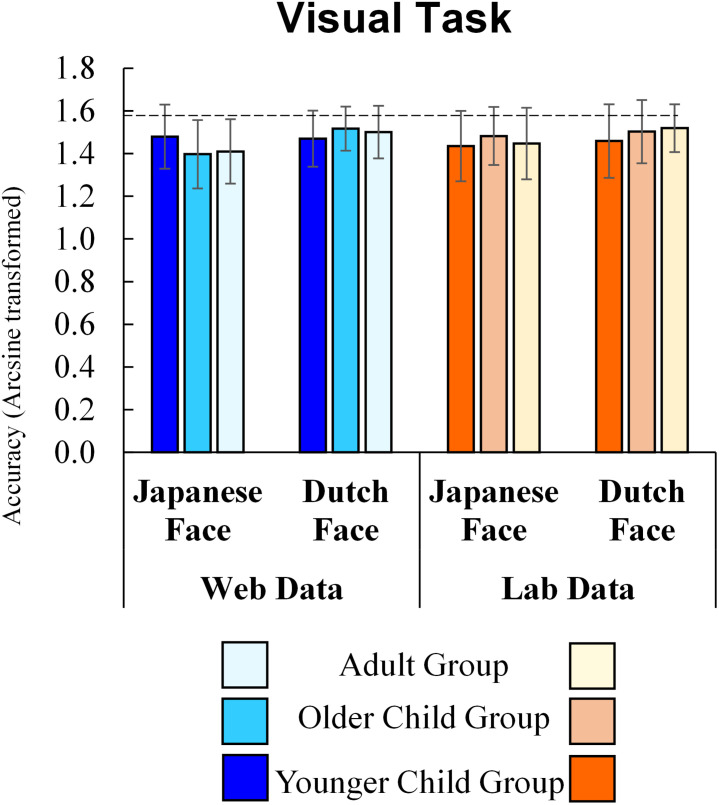
Visual task Accuracy (Error bars indicate standard deviations and a dashed line indicates a perfect score).

The results of the visual task are shown in Figure 4. In the visual task, the main effects of method (*F*(1, 257) = 0.36, *p* = 0.551, η_p_^2^ = 0.001), the interaction of method and age group (*F*(2, 257) = 1.10, *p* = 0.335, η_p_^2^ = 0.008), method and stimulus culture (*F*(1, 257) = 1.00, *p* = 0.320, η_p_^2^ = 0.004), and the second-order interaction of method, age group and stimuli (*F*(2, 257) = 1.99, *p* = 0.138, η_p_^2^ = 0.015) were not significant. Thus, the results of the visual task using online tools were not significantly different from those of the lab experiment.

Results indicated a significant main effect for stimulus culture (*F*(1, 257) = 13.92, *p* < 0.001, η_p_^2^ = 0.051), showing that participants responded correctly to the Dutch face more often than to the Japanese face. Interaction between age group and stimulus culture (*F*(2, 257) = 3.05, *p* = 0.049, η_p_^2^ = 0.023) was also significant, but the main effect for age group was not (F(2, 257) = 0.19, *p* = 0.829, η_p_^2^ = 0.001). A simple main effect analysis revealed that older children (*p* = 0.007) and adults (*p* = 0.003) responded correctly to the Dutch face more often than to the Japanese face, while younger children’s accuracy did not differ between stimulus cultures (*p* = 0.744). The accuracy for faces did not differ among age groups both for Japanese (*p* = 0.624) and Dutch stimuli (*p* = 0.165).

To further examine the effect of method on accuracy, similar to the auditory task, we conducted a Bayesian repeated measures ANOVA using JASP with default prior scales. Table 3 shows the inclusion probabilities and inclusion Bayes factor. The Bayesian ANOVA revealed that the effect of method type (BFinclusion = 0.084), the interaction effect between method and stimulus culture (BFinclusion = 0.083), the interaction effect between method and age group (BFinclusion = 0.033), and the second-order interaction of method, age group, stimulus culture, and age (BFinclusion = 0.006) were all small, supporting no effect of the difference between the web and lab experiments. Consistent with the results of classical ANOVA, the data provided moderate evidence for the effect of stimulus culture (BFinclusion = 5.893). However, the effect of the interaction between age group and stimulus culture was not informative (BFinclusion = 0.128).

**TABLE 3:** Evidence for the presence of particular effects in the accuracy of the visual task (Data averaged over all the models including/excluding a particular predictor).

	P(incl)	P(excl)	P(incl| data)	P(excl| data)	BFincl
Stimulus Culture	0.737	0.263	0.943	0.057	5.893
Age Group	0.737	0.263	0.300	0.700	0.153
Method	0.737	0.263	0.191	0.809	0.084
Stimuli × Age Group	0.316	0.684	0.056	0.944	0.128
Stimuli × Method	0.316	0.684	0.037	0.963	0.083
Method × Age Group	0.316	0.684	0.015	0.985	0.033
Stimuli × Method × Age Group	0.053	0.947	>0.001	1.000	0.006

Thus, results showed that the experiment method (web or lab) did not affect participants’ performance in either the auditory task or the visual task; that is, the web data obtained in the present study did not differ from our previously obtained lab data. That is, our method can enable researchers to obtain data that would be comparable to those of a laboratory experiment in the emotion perception tasks.

### Reaction Time

Here we reported reaction times in the online experiment. Since we did not measure reaction times in the lab experiment, we cannot compare them between methods. Moreover, we did not instruct participants to respond to each stimulus as quickly as they could. Thus, the reaction times data here are for reference only. Nevertheless, this is useful as data of online developmental experiment, and it also enable us to investigate whether we could find age difference in performance for the visual task, in which no age difference in accuracy was found due to the ceiling effect.

**FIGURE 5:**
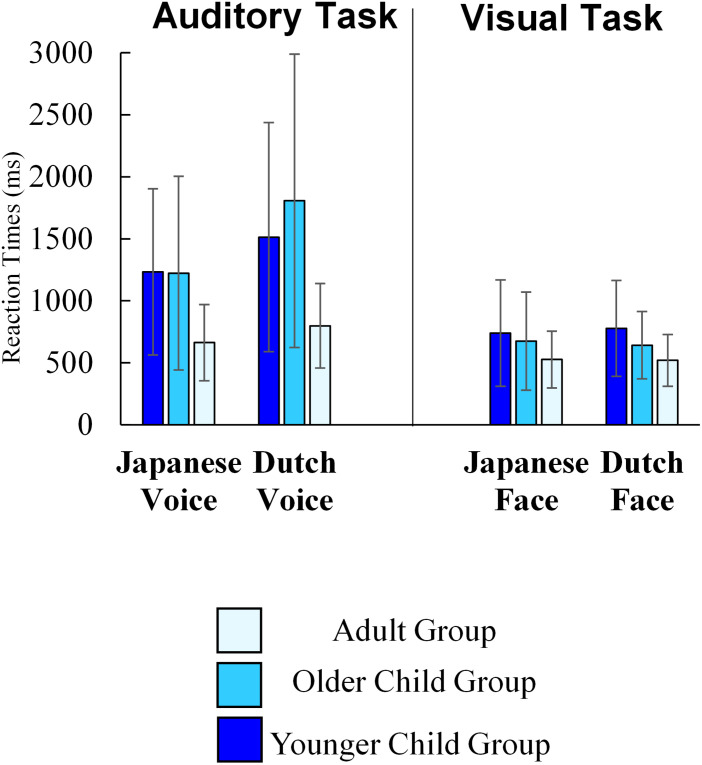
The average of participants’ reaction time in web data (Error bars indicate standard deviations).

We showed average reaction times of each task in Figure 5. We excluded outlier reaction time data (each participant’s average reaction time ±2.5 SD), while including trials in which participants pressed the wrong key, considering the following reasons. First, children’s responses classified as “incorrect responses” may include the results of their careful consideration. Second, given the age differences in accuracy, the number of correct responses, that is, the number of trials included in the analysis differed among age groups in the auditory task. We conducted a 3 (age group) × 2(stimulus culture) mixed-factorial ANOVA on reaction time for each task. In the auditory task, the main effects for age group (*F*(2, 48) = 4.91, *p* = 0.011, η_p_^2^ = 0.17) was significant. The *post hoc* analysis (Shaffer’s Modified Sequentially Rejective Bonferroni Procedure) revealed that adult group of participants responded more quickly than the younger (*p* = 0.019) and older child groups (*p* = 0.019). There was no significant difference between the younger and older child groups (*p* = 0.587). The main effect of stimulus culture was also significant (*F*(1, 48) = 27.67, *p* < 0.001, η_p_^2^ = 0.37), showing that participants responded more quickly to the Japanese voice than to the Dutch voice. Interaction between age group and stimulus culture was also significant (*F*(2, 48) = 3.91, *p* = 0.027, η_p_^2^ = 0.14). To check whether the impact of stimulus culture was different among age groups, we conducted a simple main effect analysis. The simple main effect analysis also showed that all groups yielded faster responses to the Japanese voice than in response to the Dutch voice (Younger child group: *F*(1, 20) = 6.01, *p* = 0.024; Older child group: *F*(1, 13) = 17.37, *p* = 0.011; Adult group: *F*(1, 15) = 8.51, *p* = 0.011). Moreover, a simple main effect of age was significant for both the Japanese voice (*F*(2, 48) = 4.35, *p* = 0.018) and the Dutch voice (*F*(2, 48) = 5.08, *p* < 0.001). *Post hoc* analysis (Shaffer’s Modified Sequentially Rejective Bonferroni Procedure) revealed that adult participants responded to Japanese voice faster than older children (*p* = 0.029) and younger children (*p* = 0.029), and adult participants responded to Dutch voice faster than older children (*p* = 0.011) and younger children (*p* = 0.022). There was no significant difference between the younger and older child groups for both Japanese voice (*p* = 0.962) and Dutch voice (*p* = 0.351). We conducted a one-way ANOVA on the ingroup advantage to further examine the interaction between age group and stimulus culture. This was calculated by subtracting the reaction time to Japanese voices from that of Dutch voices. The main effect of age groups was significant (*F*(2, 48) = 3.91, *p* = 0.027, η_p_^2^ = 0.01). *Post hoc* analysis (Shaffer’s Modified Sequentially Rejective Bonferroni Procedure) showed that the ingroup advantage in older children was larger than that in both adults (*p* = 0.025) and younger children (marginally significant; *p* = 0.053). The difference between younger children and adults was not significant (*p* = 0.338). Thus, the ingroup advantage based on reaction times was most salient in older children, unlike the analysis of accuracy.

In the visual task, the main effects of age group (*F*(2, 48) = 2.18, *p* = 0.124, η_p_^2^ = 0.08) and of stimulus culture (*F*(1, 48) < 0.01, *p* = 0.991, η_p_^2^ < 0.001), and the interaction between them (*F*(2, 48) = 0.84, *p* = 0.438, η_p_^2^ = 0.03) were not significant. Thus, all groups responded visual stimulus quickly and there was no difference among age groups and between stimulus culture. This is consistent with that high accuracy was observed in all age groups. Taken together, there was no differences among age groups both in accuracy and reaction times in the visual task.

## Discussion

The purpose of this study was to investigate the validity of online developmental studies through an emotion perception experiment. To that end, we conducted an experiment controlled by Gorilla Experiment Builder with child and adult participants who engaged in simple auditory and visual emotion perception tasks following communication and instruction through Zoom. As predicted, results showed no significant differences in participants’ performance between our web data results and out lab data results (Kawahara et al., 2021). In the auditory task, we found performance differences between age groups (between-subject factor) and better performance with stimuli spoken in their native language (within-subject factor). These findings were consistent with previous laboratory studies reporting performance improvements with age (Sauter et al., 2013; Chronaki et al., 2014) and superior perception of vocal emotional expression with native language stimulus (Sauter et al., 2010). In the visual task, accuracy was high and near-perfect among all age groups both in our web and lab data. To date, although online experiments have replicated laboratory experiment results with adult participants (e.g., Crump et al., 2013), developmental studies with child participants have been limited. By including child participants in our online study of emotional perception tasks, the present study adds new evidence regarding the validity of data collected in online developmental studies.

Notably, accuracy of perception of vocal expression in the lab experiment was replicated in the web experiment even though participants were not required to use a specific device and were allowed to use any earphones or headphones. The results of the present study may relieve researchers’ hesitation to conduct developmental experiments online, at least in the field of auditory emotional perception. Of course, we need additional examination to determine the suitability of online platforms for other types of auditory perception research. In tasks such as phoneme perception, judgment of speaker identities, or perceiving vocal expression from among multiple choices, participants’ responses may be affected by the devices they use (see Woods et al., 2017).

We cannot strongly conclude that online developmental research is valid for the task of perceiving facial expressions because we observed a ceiling effect; that is, performance was near perfect among all age groups in the present task. Our stimuli for the visual task were quite clear—Tanaka et al. (2010) originally created them by adding random dynamic noises to be degraded—and only two response alternatives (angry or happy) were available. The reasoning behind Kawahara et al.’s (2021) use of visual stimuli without noises was to avoid unpleasantness for the children, and so the present study followed that procedure. However, to investigate the impact of browser experiments on the presentation of visual stimuli in detail, we should conduct online studies using low intensity facial expressions, with more variety of emotions, or with smaller sized pictures in the future.

Although our main purpose was to investigate the validity of data obtained through online experiments, our data provide interesting findings on perceptual development. First, in the auditory task, the difference in the ingroup advantage was marginally significantly different between younger children and adults. That is, the ingroup advantage in younger children may be more salient than that in adults. This tendency may be related to the findings that young children prefer people who spoke their native language (e.g., Kinzler et al., 2007). Second, in the visual task, participants gave more correct responses to Dutch faces than to Japanese faces. These results are unexpected considering that previous studies have insisted on the ingroup effect in facial recognition tasks (e.g., Elfenbein and Ambady, 2002). However, more recent studies have demonstrated that Japanese raters did not show the ingroup advantage in the perception of facial expressions (Matsumoto et al., 2009; Hutchison et al., 2018). Overall, Japanese people’s facial expressions may not necessarily be perceived correctly by ingroup members. Our results of the visual task may also reflect this. Moreover, the interaction between age group and stimulus culture on accuracy was also significant in the visual task, suggesting that older children and adults responded more correctly to Dutch facial expressions than to Japanese facial expressions. These may be interesting if Japanese people judge their outgroup facial expressions more accurately compared to Japanese stimuli with age. Japanese children may come to know that Japanese people tend to conceal their true emotions (e.g., Matsumoto, 1990) and that they inhibit their facial expressions, and this may cause “outgroup advantage.” This may lead to Japanese people’s tendency to prioritize voice in audiovisual emotion perception as for Japanese stimuli shown in previous studies (Yamamoto et al., 2020; Kawahara et al., 2021). We cannot clarify this speculation based on the present study because the Bayesian ANOVA did not provide strong evidence for the interaction between age group and stimulus culture in both tasks. However, we need to investigate these perceptual developmental suggestions in the further study.

We showed new possibilities for using simple, general (not specialized for children) online tools that may enable researchers to move their laboratory studies online. However, we should clarify the limitations of the methods presented here. First, this study targeted children who could be instructed verbally and could respond by pressing keys on a keyboard. Considering that Japanese preschool children read written words from relatively early on, we did not check the level of literacy for each participant. However, checking this would be important for researchers to apply this method to children living in various environments. For studies targeting preliterate children as participants, researchers should select a video-recorder type experiment model and record participants’ oral or pointing responses. Second, we had to rely on parents’ self-reports and could not independently check children’s actual states during the browser experiment because we instructed parents to turn off their web cameras. Although we ensured that parents did not ask their children to respond in line with parents’ answers during the main trials by questionnaires, we did not have a way to confirm this was the case. Moreover, it is possible that parents would have given their children instructions without being aware of it. This could be avoided by keeping the web cameras on during the experiment. However, this could affect the quality of the presentation of the stimuli due to internet connection speed issues. These are the trade-offs, and in the present study we gave prioritized the quality of the stimuli. Such choices should be made in accordance with the aim of each study. Moreover, we should not forget the burden on parents during online experiments, and minimizing this burden should be considered when designing online experiments. In addition, as described in the section “Materials and Methods,” our web experiment procedure was not exactly the same as that of our laboratory experiment (Figure 2). Nevertheless, there were no significant differences between our web data and lab data in the present study, which suggests that the difference in procedure does not have a critical impact on the results in the experiments investigating the development of emotion perception.

As an online experiment research, the procedure of the present study has two particularities. First, child participants’ parents take on the “experimenter” role. Second, researchers and participants communicate with each other through video chat before the tasks. Previous online psychological experiment studies with adults have pointed out high dropout rates (e.g., Reips, 2002), large variances due to various environments among participants, and difficulties in a between-subject design study. On the contrary, it is worth noting that the methods we adopted resulted in very few cases of participant data being excluded. Moreover, the variance of performance seemed to be similar to that of the lab experiment even though participants used their laptops and earphones (headphones). Our only requests before the experiment were to enter the video chat at the appointed time and to prepare earphones or headphones. Considering the effort involved in making an appointment with each participant, and in instructing both parents and child participants to proceed with the experiments, our method does not have benefits such as large data collecting in a short period, unlike usual crowdsourced online experiments. Nevertheless, the results of the present study suggest that this effort can reduce issues associated with online research, such as a dropout rate and variance of data. Given that even adult participants engaged in tasks seriously without an experimenter, video chat communication before the main experiment may be specifically effective. Even though our method does not have the aforementioned benefits associated with crowdsourced online research, we regard its biggest advantage to be the fact that both experimenters and participants are not affected by geographical constraints. In fact, as long as they have an internet connection, researchers can conduct studies with people living in various countries and continue to collect data even under a pandemic.

We should note that the reproducibility of results in online experiments may depend on indices. We used the rate of correct responses, and we did not compare other indices such as reaction times or fixations. Given that our web data showed that all age groups responded more quickly to the Japanese stimuli in the auditory task, reaction time may be used even in online experiments. However, unlike the results for accuracy, we did not find age differences between child groups for reaction times. Moreover, while we observed a salient ingroup advantage in younger children compared with adults (although the tendency was marginally significant) for accuracy, this was not reflected in reaction times. Rather, for reaction times, we found that older children’s ingroup advantage was more salient than the other two age groups. Since we do not have reaction time data of our lab experiment, it remains unclear whether such tendency is observed also in a lab experiment or is unique to an online experiment. As a limitation of online research, one previous study pointed out the difficulty in controlling a short presentation of stimuli such as a masked priming procedure (Crump et al., 2013). Another study investigating the contrast threshold reported a high rate of data exclusions due to each participant’s experimental environment (Sasaki and Yamada, 2019). Further studies are needed to examine which indices and tasks are adequate for online experiments. Despite the limitations, we demonstrated that online experiments are useful for child research using auditory and visual (movie) stimuli. Combinations of online tools will lead researchers to new developmental research styles. Moreover, due to the validity of this online research using unimodal auditory and visual stimuli, application to future audiovisual perception research is expected.

## Data Availability Statement

The raw data supporting the conclusions of this article will be made available by the authors, without undue reservation.

## Ethics Statement

The studies involving human participants were reviewed and approved by Tokyo Woman’s Christian University Research Ethics Committee. Written informed consent or digital informed consent to participate in this study was provided by the participants’ legal guardian/next of kin.

## Author Contributions

HY was involved in designing the web experiment programs, collecting the data in the web experiment, analyzing the data, and drafting the manuscript. MK was involved in collecting the data and designing programs in the laboratory experiment. AT was involved in the creation of stimuli. All authors were involved in the experimental design, interpretation of the results, revised, and approved the final version of the manuscript.

## Conflict of Interest

The authors declare that the research was conducted in the absence of any commercial or financial relationships that could be construed as a potential conflict of interest.

## Publisher’s Note

All claims expressed in this article are solely those of the authors and do not necessarily represent those of their affiliated organizations, or those of the publisher, the editors and the reviewers. Any product that may be evaluated in this article, or claim that may be made by its manufacturer, is not guaranteed or endorsed by the publisher.

## Footnotes

1
https://zoom.us/

2
https://gorilla.sc/

3 Anwyl-Irvine et al. (2020) showed that child participants had completed a flanker task created with Gorilla Experiment Builder (the youngest participant in the final sample was 4.38 years old) and revealed the development of the children’s performance. This was not a remote online study because participation in the experiment took place in a laboratory setting with an experimenter, not in participants’ homes. Nonetheless, the results do suggest that even child participants can engage in cognitive tasks controlled by this platform.

4
https://crowdworks.co.jp/

5 According to EMFACS (Friesen and Ekman, 1984), the activation of AU 17 is related to negative expressions such as distress and rage. Considering this fact, it is possible that the activation of AU 17 leads to the judgment of facial expressions as negative. Nevertheless, for stimuli in the present study (Kawahara et al., 2021), we confirmed that both Japanese and Dutch adult participants’ accuracy of perceiving angry faces did not differ between stimulus cultures. Therefore, we consider that the frequency of activation in AU 17 did not significantly impact participants’ judgment in the present study.

6 We used response alternatives written in hiragana characters (angry: おこっている [okotteiru]; happy: よろこんでいる [yorokondeiru]) because both young children and adults can read them easily. According to previous studies on Japanese children’s literacy, more than half of 3–4-year-olds could read most of hiragana characters (Kakihana et al., 2009), and about 90% of children in 5-year-old classes could read them (Ota et al., 2018) without formal education. Since Japanese hiragana characters have high transparency (each hiragana character corresponds to a syllable in Japanese phonology), Japanese children who know hiragana characters can be assumed to also read words. Considering Japanese children’s literacy, we inferred that the participants in this study could read response alternatives.

7 We did it because it is easier for native Japanese speaking adults to read sentences such as instructions that use both kanji and hiragana than sentences written in hiragana characters only.
